# Patients with Inflammatory Bowel Disease Are at an Increased Risk of Parkinson’s Disease: A South Korean Nationwide Population-Based Study

**DOI:** 10.3390/jcm8081191

**Published:** 2019-08-08

**Authors:** Seona Park, Jihye Kim, Jaeyoung Chun, Kyungdo Han, Hosim Soh, Eun Ae Kang, Hyun Jung Lee, Jong Pil Im, Joo Sung Kim

**Affiliations:** 1 Department of Internal Medicine and Liver Research Institute, Seoul National University College of Medicine, Seoul 03080, Korea; 2 Department of Internal Medicine, CHA Gangnam Medical Center, CHA University, Seoul 06135, Korea; 3 Department of Internal Medicine, Gangnam Severance Hospital, Yonsei University College of Medicine, Seoul 06229, Korea; 4 Department of Biostatistics, College of Medicine, The Catholic University of Korea, Seoul 06591, Korea

**Keywords:** Claims data, Crohn’s disease, inflammatory bowel disease, Parkinson’s disease, ulcerative colitis

## Abstract

**Background and Aims:** It is not known whether inflammatory bowel disease (IBD) enhances the risk of Parkinson’s disease (PD) or whether PD diagnosis is the result of increased health care use. We determined the risk of developing PD among patients with IBD in terms of health care and medication use. **Methods:** A nationwide population-based study was conducted using claims data from the Korean National Health care Insurance service. From 2010 to 2013, patients with Crohn’s disease (CD) and ulcerative colitis (UC) were identified through both International Classification of Disease, Tenth Revision (ICD-10) and national rare intractable disease (RID) registration program codes. We compared 38,861 IBD patients with age and sex-matched non-IBD individuals at a ratio of 1:3. Patients with newly diagnosed PD were identified through both ICD-10 and RID codes. **Results:** The incidence of PD among patients with IBD was 49 per 100,000 person-years. The risk of developing PD in patients with IBD was significantly higher than controls even after adjustment for health care use (adjusted hazard ratio (aHR), 1.87; *P* < 0.001). Compared to controls, the risk of PD was significantly higher in patients with CD (aHR, 2.23; *P* = 0.023) and UC (aHR, 1.85; *P* < 0.001). Corticosteroid use showed a preventive effect on developing PD in patients with CD (aHR 0.08; *P* < 0.001), but not UC (aHR, 0.75; *P* = 0.213). Among 2110 patients receiving anti-tumor necrosis factor (anti-TNF), none of the treated patients experienced PD during 9950 person-years. **Conclusion:** Patients with IBD are at an increased risk of PD, regardless of health care use. Corticosteroid and anti-TNF use may prevent PD in patients with IBD.

## 1. Introduction

Parkinson’s disease (PD) is the second most common neurodegenerative disorder and is characterized by the premature loss of dopaminergic neurons in the substantia nigra and abnormal aggregates of the α-synuclein protein, affecting 1% of people over 60 years of age. The disease progresses slowly and causes motor dysfunction, such as bradykinesia, resting tremor, rigidity and postural instability, and non-motor features, such as hyposmia, sleep disturbances, depression, constipation, and other dysautonomic symptoms [1]. Most epidemiological studies have shown that PD increases mortality hazard ratios by 1.5–2.7 times compared to the general population [2]. In addition, 25–40% of patients with PD eventually develop dementia, which also contributes to reduced life expectancy [3,4]. Although numerous risk factors, including oxidative stress, environmental toxins, and some genetic mutations, are known to be associated with PD, the pathophysiology of neurodegeneration in PD has not been fully elucidated [5,6]. Recently, accumulating evidence has demonstrated that systemic inflammation is closely linked to the pathogenesis of PD, which suggests that chronic inflammation in peripheral organs may contribute to neurodegeneration in PD by altering blood–brain barrier permeability [7,8]. Peripheral cytokines, such as interleukin (IL)-6, tumor necrosis factor (TNF), IL-1β, IL-2, IL-10 and C-reactive protein, are increased in patients with PD [9,10]. As such, human studies have shown that a variety of chronic inflammatory diseases including autoimmune rheumatoid arthritis [11], Sjögren syndrome [12], and periodontal inflammatory disease [13] increase the risk of PD development.

Inflammatory bowel disease (IBD), which includes Crohn’s disease (CD) and ulcerative colitis (UC), is a chronic, relapsing inflammatory disease of the intestine [14]. Dynamic interactions between genetic predisposition, environmental, and immunological factors contribute to the pathogenesis of IBD [15,16,17]. The dysbiosis of gut microbiota and destruction of the epithelial barrier affect the initiation and perpetuation of chronic inflammation in the gut [18,19]. In addition, psycho-neuro-endocrine-immune modulation through the brain–gut axis may play an important role in the pathogenesis of IBD [20]. Growing awareness of communication between the intestinal environment and central nervous system activity through the brain–gut axis has led to the hypothesis that chronic intestinal inflammation may result in neurodegeneration in PD [21]. It has been confirmed that IBD and PD share common genetic risk profiles, such as *NOD2*, *LRRK2* and *MAPT* genes [22,23,24,25,26].

Several recent epidemiologic studies [27,28,29,30,31,32,33] have been conducted to determine whether IBD increases the risk of PD based on common biologic and genetic mechanisms described in the pathogenesis of IBD and PD. However, there is still controversy regarding the risk of PD in patients with IBD because increased health care use following a diagnosis of IBD is likely to act as a surveillance bias in interpreting a causal relationship between IBD and the risk of PD [34,35]. In addition, whether therapeutic agents for treating IBD lower the risk of developing PD remains unknown. The aim of this nationwide population-based study was to determine the risk of developing PD among patients with IBD compared to the general population in terms of health care use and concomitant medication for treating IBD.

## 2. Material and Methods

### 2.1. Data Source

We conducted a retrospective population-based cohort study using a database provided by the National Health Insurance Service (NHIS) in South Korea. The NHIS is a single payment program established by the Korean government. Approximately 52 million residents in Korea are obliged by law to enroll in NHIS, and the health care provider must submit the required claims data to receive medical reimbursement. Therefore, all relevant data are accumulated in the NHIS database [36]. The NHIS database contains information on personal demographics, hospitalization and outpatient medical use, medications, medical procedures and diagnoses identified by International Classification of Disease, Tenth Revision (ICD-10) codes. NHIS has established a rare intractable disease (RID) enrollment program to strengthen medical reimbursement by reducing the economic burden of treating RIDs, such as PD and IBD. To enroll patients with RIDs in this program, qualified physicians have to evaluate whether the diseases meet the diagnostic criteria for each RID. When registered in the RID program, a special diagnostic code (V code) is assigned in addition to the ICD-10 code. For patients with IBD, the V code is provided only if they meet specific comprehensive diagnostic criteria, including imaging, histologic, clinical and endoscopic findings. This study protocol was exempt from Seoul National University Hospital Institutional Review Board (H-1703-107-840).

### 2.2. Study Population

In this study, we included 38,861 patients with IBD who enrolled with both ICD-10 and V codes from January 2010 to December 2013 in the NHIS database. CD patients were identified using both ICD-10 K50 and V130 codes, and UC patients were identified using both ICD-10 K51 and V131 codes. As described in previous studies [37,38,39,40,41,42], the diagnostic accuracy of IBD using both the V code and ICD-10 codes has been validated. In brief, the diagnostic sensitivity of CD and UC was found to be 94.5 and 96.4%, respectively. In addition, the IBD cohort was divided into the incident IBD group, which was newly registered with both ICD-10 and V codes from January 2010 to December 2013 (patients without a history of IBD from January 2005 to inclusion date), and prevalent IBD group, for which the IBD code was registered between 2005 and inclusion date, with reimbursement for IBD between 2010 and 2013. A total of 116,583 age- and sex-matched individuals without IBD were randomly selected as non-IBD controls at a 1:3 ratio. Individuals with other immune-mediated diseases were not excluded from the non-IBD controls.

### 2.3. Data Collection

In addition to demographics (age, sex, place of residence, and income level), information about the drugs used to treat IBD, including corticosteroids, immunomodulators (azathioprine/6-mercaptopurine and methotrexate), and anti-TNF agents (infliximab, adalimumab and golimumab), in both IBD and non-IBD groups was collected. Medication use was defined as a prescription issued within 1 year following IBD diagnosis. We also collected information on comorbidities, including hypertension (defined as ICD-10 codes (I10–13, I15) with antihypertensive agents), diabetes mellitus (DM; defined as ICD-10 codes (E11–14) with antihyperglycemic agents), dyslipidemia (defined as ICD-10 code (E78) with antihyperlipidemic agents), depression (defined as ICD-10 code (F32–34)), myocardial infarction (defined as ICD-10 code (I21–22) with hospitalization), stroke (defined as ICD-10 code (I63–64) with hospitalization and brain imaging with computed tomography or magnetic resonance imaging), and ischemic heart disease (defined as ICD-10 code (I20–25) with hospitalization), as described previously [39,42,43,44]. Health care use was defined as a total number of inpatient and/or outpatient visits within 1 year after IBD diagnosis or the inclusion date.

### 2.4. Study Endpoint

PD was defined as a patient assigned both ICD-10 (G20) and V codes (V124), as described in a previous study [45]. The ICD-10 G21 code (secondary parkinsonism) was not used to detect individuals with idiopathic PD. In general, PD is diagnosed based on the clinical characteristics, and the United Kingdom (UK) Parkinson’s Disease Society Brain Bank criteria is used in practice [5]. The criteria for the V124 code are identical to the UK Parkinson’s Disease Society Brain Bank diagnostic criteria as follows: (1) The diagnostic criteria for PD include mild or worse bradykinesia and at least 1 of the following: muscular rigidity, rest tremor and postural instability. (2) The exclusion criteria for PD are a history of strokes, head injury, definite encephalitis, drug adverse events and hypoxia. (3) The supportive prospective positive criteria for PD include 3 or more of the following conditions in combination with those from (1), which are required for the diagnosis of definite PD: unilateral onset, presence of rest tremor, progressive disorder, persistent asymmetry affecting the side of onset the most, excellent response to levodopa, severe levodopa-induced chorea, levodopa response for 5 years or more and clinical course of 10 years or more [45]. The IBD cohort and non-IBD controls were followed up until the occurrence of PD or December 2015. The primary endpoint was newly developed PD during the follow-up period.

### 2.5. Statistical Analysis

Baseline demographics, comorbidities and medications in the IBD cohort and non-IBD control were compared. Differences between the two groups were assessed with independent t-tests for continuous variables and χ^2^ tests for categorical variables. The incidence of PD was calculated as the number of events per 100,000 person-years in each group. Cox proportional hazard regression models were used to show the effect of IBD on the risk of PD. Results were expressed as hazard ratio (HR) and 95% confidence interval (CI). In multivariable models, age, sex, place of residence, income level, comorbidities as well as health care use were adjusted. The cumulative incidence of PD in the IBD and non-IBD groups was compared using the Kaplan–Meier method and log-rank test. A *P* Value < 0.05 was considered statistically significant. R program version 3.4.3 (The R Foundation for Statistical Computing, Vienna, Austria, http://www.R-project.org) and SAS version 9.2 (SAS Institute Inc., Cary, NC, USA) for Windows were used for statistical analysis.

## 3. Results

### 3.1. Baseline Characteristics of the Study Population

From January 2010 to December 2013, a total of 38,861 and 116,583 individuals were enrolled in the IBD and non-IBD groups, respectively, matched by age and sex. The mean age was 39.9 years and 61.2% were male. The study participants aged 60 and older were 14.0, 5.6, and 18.1% in IBD, CD, and UC groups, respectively. The IBD cohort was significantly associated with a lower proportion of rural residents (*P* < 0.001) and income levels below 20% (*P* < 0.001) compared to non-IBD control. The prevalence of DM (*P* < 0.001), hypertension (*P* < 0.001), and dyslipidemia (*P* = 0.029) was significantly lower in the IBD cohort compared to controls. In contrast, myocardial infarction (*P* < 0.001), stroke (*P* = 0.023), ischemic heart disease (*P* < 0.001), and depression (*P* < 0.001) in the IBD cohorts was significantly more prevalent than in the non-IBD controls. The frequency of health care use was significantly higher in the IBD cohort compared to controls (20.0 visits/year vs. 11.9 visits/year; *P* < 0.001). Among the medications used to treat IBD, corticosteroids, immunomodulators and anti-TNF agents were prescribed in 57.4, 26.8 and 5.4% of the IBD cohort, respectively, and were used more frequently compared to non-IBD controls (Table 1).

### 3.2. Incidence and Risk of Parkinson’s Disease in Patients with Inflammatory Bowel Disease

**Figure 1:**
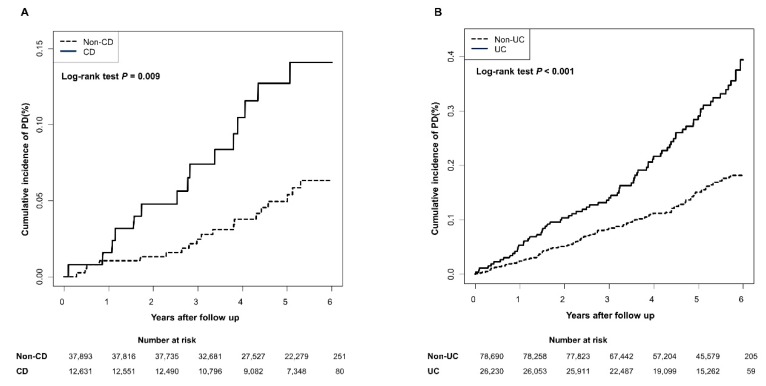
Kaplan–Meier plots show the cumulative incidence of Parkinson’s disease in patients with inflammatory bowel disease. (**A**) Patients with Crohn’s disease; (**B**) Patients with ulcerative colitis. Abbreviations: CD, Crohn’s disease; PD, Parkinson’s disease; UC, ulcerative colitis.

Of the 38,861 patients in the IBD cohort, 16,620 were in the incident IBD group and 22,241 were in the prevalent IBD group. During a mean follow-up of 4.9 years, 92 patients (0.24%) in the IBD cohort and 134 patients (0.11%) in the non-IBD control group developed PD. The mean age at diagnosis of PD was significantly lower in the IBD patients than in the non-IBD controls (60.9 vs. 64.1 years; *P* = 0.036). The mean age at diagnosis of PD in those with CD was significantly lower compared to the controls (53.7 vs. 64.9 years; *P* = 0.014), but there was no significant difference in the mean age at diagnosis of PD between UC and control groups (62.3 vs. 63.9 years; *P* = 0.305). The cumulative incidence of PD in patients with CD and UC was significantly higher compared to the control groups (Figure 1A,B).

The incidence and risk of PD in the IBD cohort was significantly higher than in non-IBD controls after adjustment for age, sex, place of residence, income level, comorbidities including DM, hypertension, dyslipidemia, depression, stroke, myocardial infarction and ischemic heart disease, and number of health care visits (49 vs. 24 per 100,000 person-years; adjusted HR, 1.87; 95% CI, 1.43–2.44; *P* < 0.001 in Model 3) (Table 2).

The incidence and risk of PD was significantly higher than in non-IBD controls when the IBD cohort was divided into the incident IBD group (38 vs. 24 per 100,000 person-years; adjusted HR, 1.58; 95% CI, 1.02–2.44; *P* = 0.039 in Model 3) and the prevalent IBD group (54 vs. 24 per 100,000 person-years; adjusted HR, 2.00; 95% CI, 1.49–2.70; *P* < 0.001 in Model 3). In patients with CD, the incidence and risk of PD was significantly higher compared to controls (24 vs. 10 per 100,000 person-years; adjusted HR, 2.23; 95% CI, 1.12–4.45; *P* = 0.023 in Model 3). Among patients in the prevalent CD groups, the incidence and risk of PD was also significantly higher compared to controls (incident CD group: adjusted HR, 1.36; 95% CI, 0.45–4.07; *P* = 0.587; prevalent CD group: adjusted HR, 2.91; 95% CI, 1.36–6.24; *P* = 0.006 in Model 3). In patients with UC, the incidence and risk of PD was also significantly higher compared to controls (60 vs. 30 per 100,000 person-years; adjusted HR, 1.85; 95% CI, 1.38–2.48; *P* < 0.001 in Model 3). Among patients in both incident and prevalent UC groups, the risk of PD was also significantly higher compared to controls (incident UC group: adjusted HR, 1.68; 95% CI, 1.05–2.69; *P* = 0.031; prevalent UC group: adjusted HR, 1.93; 95% CI, 1.40–2.67; *P* < 0.001 in Model 3).

When the comparative risks of PD in the study population were stratified by age at 60, IBD patients had significantly increased risk of PD compared to controls, regardless of age (aged <60 years: adjusted HR, 2.63; 95% CI, 1.57–4.41; *P* < 0.001 in Table S2; aged ≥60 years: adjusted HR, 1.63; 95% CI, 1.19–2.24; *P* = 0.003 in Table S3). However, there was no statistically significant difference in the risk of PD between CD and control groups among individuals aged ≥60 years (Table S3).

### 3.3. Incidence and Risk of Parkinson’s Disease According to Medication Use

The incidence and risk of PD among CD patients, but not UC patients, who were treated with corticosteroids was significantly lower than individuals who were not treated with corticosteroids (11 vs. 41 per 100,000 person-years; adjusted HR 0.08; 95% CI, 0.02–0.33; *P* < 0.001 in Model 2) (Table 3). In terms of immunomodulator use, there was no significant difference in the incidence of PD among patients with CD and UC. Of the 2110 patients who received anti-TNF agents, no patient experienced PD during 9950 person-years (Table 3). In non-IBD controls, there was no significant difference in the risk of PD according to medication use (Table 3).

### 3.4. Subgroup Analysis

**Figure 2:**
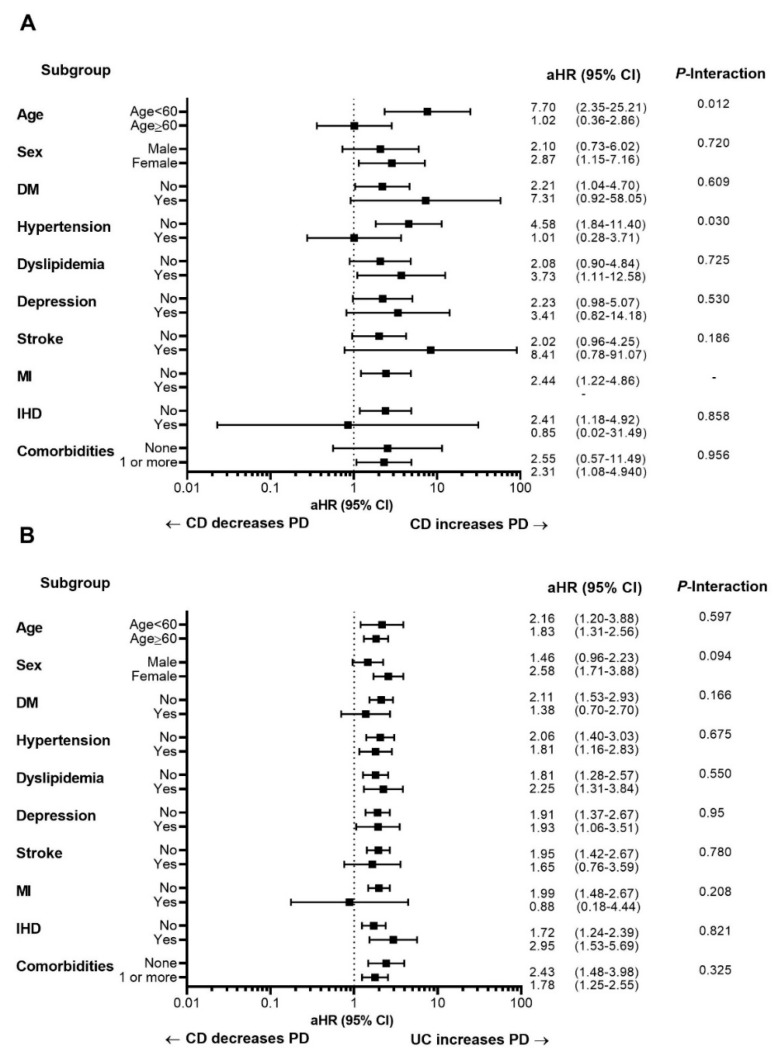
Analysis of Parkinson’s disease in subgroups of patients with inflammatory bowel disease. The cohort was divided into patients with (**A**) Crohn’s disease or (**B**) ulcerative colitis. Subgroups included younger/older age, male/female sex, patients with/without DM, hypertension, dyslipidemia, depression, stroke, myocardial infarction, ischemic heart disease, and patients with/without comorbidities. Abbreviations: CD, Crohn’s disease; CI, confidence interval; DM, diabetes mellitus; aHR, adjusted hazard ratio; IHD, ischemic heart disease; MI, myocardial infarction; PD, Parkinson’s disease; UC, ulcerative colitis.

In this subgroup analysis, we evaluated differences in the risk of PD according to age, sex, and comorbidities of patients with CD and UC after adjustment for age, sex, place of residence, income level, comorbidities including DM, hypertension, dyslipidemia, depression, stroke, myocardial infarction and ischemic heart disease. The impact of CD on developing PD was more prominent in patients less than 60 years of age (adjusted HR, 7.70 vs. 1.02; interaction *P* value 0.012) and without hypertension (adjusted HR, 4.58 vs. 1.01; interaction *P* value 0.030) (Figure 2A). In contrast, there was no significant difference in the risk of PD according to the subgroup consisting of patients with UC (Figure 2B).

## 4. Discussion

In this nationwide population-based study of approximately 160,000 Korean individuals, including 39,000 patients with IBD during a mean follow-up of 5 years, we demonstrated that patients with IBD were 1.9 times significantly more likely to develop PD compared to non-IBD age- and sex-matched controls, even after adjustment for health care visits and comorbidities. Of the IBD subtypes, CD and UC patients were at 2.2- and 1.9-times higher risk for PD than controls, respectively.

In a literature review, five retrospective studies compared the risks of developing PD between IBD and non-IBD groups (Table S1) [28,29,30,32,33,46]. In a case-control study from the United States [29], PD was inversely associated with both CD and UC compared to non-PD controls after adjustment for health care use, but only patients with PD over 65 years were included. In contrast, in a US cohort study [33], the risk of PD was significantly higher in CD and UC patients compared to controls. In a Taiwanese cohort study [32], the risk of PD was also 40% significantly higher in CD, but not UC, compared to controls. However, both studies did not consider surveillance bias effects on the risk of PD in IBD patients. Recently, a Swedish cohort study [30] reported that the risk of PD was 1.3 times higher in UC compared to controls, but the increased risk of PD was not significant after adjustment for health care visits. These findings have led to debates over whether IBD patients are exposed to an increased risk of PD because IBD leads to increased health care uses. In contrast, a Danish cohort study [28] showed a 22% increased risk of PD in IBD, which was also significant after adjustment for number of health care visits (HR, 1.49; 95% CI, 1.34–1.66) [46].

In the present study, the increased risks of developing PD among IBD patients compared to controls were statistically significant even after adjustment for health care visits, which is consistent with the results of the Danish study [28,46]. The risks of developing PD among IBD patients were the highest compared to all previous epidemiologic studies, even after adjustment for health care visits. It should be noted that the mean age of the study population was 45 years or over in the other studies. Aging is a crucial risk factor for developing PD [47]. Older mean age at the baseline is related to higher risks of developing PD in the non-IBD controls. In this study, the mean age of the study population was 40 years, and 92% of the subjects were under the age of 45. In addition, the comparative risk of developing PD in patients with CD compared to controls was significant only among individuals younger than 60 years of age. In terms of UC, the comparative risks for PD were significant compared to controls regardless of age subgroups, but the adjusted HR decreased more in individuals greater than or equal to 60 years of age than in those less than 60. Considering the confounding effect of age on the occurrence of PD, IBD itself is an independent risk factor for developing PD. Moreover, it should be noted that the age at diagnosis of PD in the IBD patients was significantly lower compared to the controls. Especially, the comorbid PD was diagnosed before the age of 60 in most CD patients. Given that IBD is a chronic inflammatory disorder occurring most commonly during adolescence and young adulthood, physicians should be aware of the potential risk for PD in patients with IBD.

Recent evidence has shown that inflammation plays a crucial role in neuro-degenerative processes of PD [48,49]. Active peripheral inflammation as well as deregulation of the inflammatory pathway due to genetic vulnerability contributes to the initiation and progression of PD. In this study, steroid therapy reduced the risk of developing PD by 92% among CD patients. Synthetic glucocorticoids act as potent anti-inflammatory drugs, and previous studies have reported that dexamethasone plays a neuroprotective role in animal PD models [50,51,52]. Moreover, there were no occurrences of PD among 2110 patients with IBD who received anti-TNF agents during approximately 10,000 person-years. This is consistent with the results of previous studies on the preventive effects of anti-TNF agents on development of PD [30,33]. TNF-α levels were increased in the substantia nigra and striatum of the 6-hydroxydopamine-injected area of hemiparkinsonian rats [53]. The expression levels of TNF-α and TNF-α receptor R1 were also enhanced in the nigrostriatal dopamine regions and ventricular cerebrospinal fluid of patients with PD, which may be responsible for degeneration of dopaminergic neurons in the substantia nigra pars compacta and loss of nerve terminals with dopamine deficiency in the striatum, leading to development of PD [53]. TNF-α contributes to neuroinflammation on passing through the blood–brain barrier [8,54,55]. Anti-TNF agents are blocked by the blood–brain barrier, but their effects on peripheral inflammation are likely to reduce neuroinflammation and the risk of PD development. A previous study reported that peripheral TNF-α modulates amyloid pathology by regulating blood-derived immune cells trafficking in a transgenic mouse model of Alzheimer’s disease [56]. Recently, a prospective cohort study demonstrated that anti-TNF agents shifted the diversity of gut microbiota in patients with IBD toward that of healthy individuals [57], suggesting that anti-TNF agents might prevent neuroinflammation via modulating the gut microbiota in the pathogenesis of neurodegenerative diseases.

There are some limitations to this study due to its retrospective design. First, we could not determine the risk of developing PD in IBD based on disease severity from the claims data. Interestingly, IBD patients receiving steroid or anti-TNF agents who may have had severe inflammatory disease showed a significantly reduced risk of developing PD compared to those with mild disease. This suggests that the impacts of steroids and anti-TNF agents outweigh the effects of intestinal inflammation on the occurrence of PD among IBD patients. Second, the risks of developing PD in IBD could not be adjusted for some potential confounding factors, such as lifestyle or personal health behaviors, including tobacco smoking, coffee drinking, and alcohol consumption [5]. The patterns of personal behavior might be different between IBD and non-IBD individuals. Third, the impacts of genetic risk profiles such as *NOD2*, *LRRK2* and *MAPT* on PD development in IBD could also not be evaluated due to the study design and data availability. Fourth, how alterations in gut microbiota composition influence the initiation of neurodegeneration in PD could not be evaluated. A shift in gut microbiota composition together with the impacts of medications, lifestyle, diet and aging may play a crucial role in the pathogenesis of PD by facilitating the ascending neurodegenerative spread of α-synuclein aggregates from the enteric nervous system to the brain [58]. Braak H. hypothesized that the pathological α-synuclein spreads via the vagus nerve to the brain leading to the development of PD [59,60], which has been recently demonstrated in a mouse model of PD [61]. Colonic inflammation in marmosets was related to the pathologic phosphorylation of α-synuclein in the colonic myenteric ganglia, which suggests that colitis may trigger the neurodegenerative spread of pathologic α-synuclein [62]. Further investigations are needed to determine how changes in the gut microenvironment contribute to the neurodegenerative pathway in human. An understanding of the attributes of gut microenvironment changes in PD may lead to targeted prevention aimed at modulating the gut microbiota. Fifth, we could not assess the risk of PD among IBD patients based on the treatment response against IBD because the medications for IBD were defined as prescription-only medications within 1 year following the diagnosis, taking into account a relatively short follow-up period. Finally, the mean follow-up period of approximately 5 years may be short to fully evaluate the significantly increased risks of PD in IBD patients, especially in incident CD patients, who showed the lowest number of PD development during the shortest observed period (only four cases per 22,000 person-years) among all subgroups. Further long-term observational studies are required to elucidate the cumulative risks of the neurodegenerative disease, mostly involving the elderly, among IBD patients after diagnosis.

## 5. Conclusions

Patients with IBD had a significantly higher risk of developing PD compared to non-IBD individuals, regardless of health care use. Therefore, patients with IBD should be aware of the potential risks for comorbid PD. Corticosteroid and anti-TNF use may prevent the occurrence of PD in patients with IBD.

## Figures and Tables

**Table 1:** Baseline characteristics of the study population.

	IBD			CD			UC		
	Non-IBD Control (*n* = 116,583)	IBD Cohort (*n* = 38,861)	*P* Value	Non-CD Control (*n* = 37,893)	CD Cohort (*n* = 12,631)	*P* Value	Non-UC Control (*n* = 78,690)	UC Cohort (*n* = 26,230)	*P* Value
Age, years *	39.91 ± 16.62	39.91 ± 16.62	-	30.74 ± 14.56	30.74 ± 14.56	-	44.33 ± 15.72	44.33 ± 15.72	-
≥60 ^‡^	16,335 (14.0)	5445 (14.0)	-	2130 (5.6)	710 (5.6)	-	14,205 (18.1)	4735 (18.1)	-
Male gender ^‡^	71,292 (61.2)	23,764 (61.2)	-	26,580 (70.1)	8860 (70.1)	-	44,712 (56.8)	14,904 (56.8)	-
Rural residence ^‡^	62,113 (53.3)	19,328 (49.7)	<0.001	20,117 (53.1)	6186 (49.0)	<0.001	41,996 (53.4)	13,142 (50.1)	<0.001
Lowest income 20% ^†,^^‡^	27,195 (23.3)	7687 (19.8)	<0.001	8785 (23.2)	2668 (21.1)	<0.001	18,410 (23.4)	5019 (19.1)	<0.001
Comorbidities									
DM ^‡^	5786 (5.0)	1610 (4.1)	<0.001	867 (2.3)	280 (2.2)	0.642	4919 (6.3)	1330 (5.1)	<0.001
Hypertension ^‡^	14,911 (12.8)	4503 (11.6)	<0.001	2312 (6.1)	684 (5.4)	0.005	12,599 (16.0)	3819 (14.6)	<0.001
Dyslipidemia ^‡^	8621 (7.4)	2744 (7.1)	0.029	1388 (3.7)	389 (3.1)	0.002	7233 (9.2)	2355 (9.0)	0.299
Depression ^‡^	3535 (3.0)	2336 (6.0)	<0.001	760 (2.0)	690 (5.5)	<0.001	2775 (3.5)	1646 (6.3)	<0.001
Ischemic heart disease ^‡^	4028(3.5)	1764 (4.6)	<0.001	631 (1.7)	392 (3.1)	<0.001	3397 (4.3)	1372 (5.2)	<0.001
History of myocardial infarction ^‡^	458 (0.4)	235 (0.6)	< 0.001	73 (0.2)	68 (0.5)	<0.001	385 (0.5)	167 (0.6)	0.004
History of stroke ^‡^	1752 (1.5)	648 (1.8)	0.023	241 (0.6)	129 (1.0)	<0.001	1,511 (1.9)	519 (2.0)	0.552
Medications for IBD									
Corticosteroids ^‡^	37,450 (32.1)	22,321 (57.4)	< 0.001	10,793 (28.5)	7417 (58.7)	<0.001	26,657 (33.9)	14,904 (56.8)	<0.001
Immunomodulators ^‡^	373 (0.3)	10,405 (26.8)	< 0.001	92 (0.2)	7008 (55.5)	<0.001	281 (0.4)	3397 (13.0)	<0.001
Anti-TNF ^‡^	28 (0.0)	2110 (5.4)	< 0.001	11 (0.0)	1665 (13.2)	<0.001	17 (0.0)	445 (1.7)	<0.001
Follow-up period, years *	4.88 ± 1.27	4.87 ± 1.29	0.201	4.90 ± 1.27	4.86 ± 1.31	0.005	4.88 ± 1.28	4.88 ± 1.28	0.704
Health care visits *	11.86 ± 17.84	20.0 ± 20.7	< 0.001	8.43 ± 13.73	17.56 ± 18.23	<0.001	13.51 ± 19.3	21.17 ± 21.69	<0.001

CD, Crohn’s disease; DM, diabetic mellitus; IBD, inflammatory bowel disease; TNF, tumor necrosis factor; UC, ulcerative colitis. * Mean ± SD. ^†^ Defined as patients whose income was in the bottom 20%. ^‡^ Number (%).

**Table 2:** Incidence and risk of Parkinson’s disease in patients with inflammatory bowel disease.

	Total No.	PD Cases	Person-Years (y)	PD Incidence (/100,000 Person-Years)	Age at PD Diagnosis, Years *	Model 1 ^†^ HR (95% CI)	*P* Value	Model 2 ^‡^ HR (95% CI)	*P* Value	Model 3 ^§^ HR (95% CI)	*P* Value
**Total IBD**							<0.001		<0.001		<0.001
Control	116,583	134	569,360	24	64.1 ± 10.2	1 (Ref.)		1 (Ref.)		1 (Ref.)	
Case	38,861	92	189,415	49	60.9 ± 12.1	2.07 (1.59–2.70)		1.95 (1.49–2.54)		1.87 (1.43–2.44)	
**IBD subgroup**											
Incident	16,620	25	65,692	38	61.8 ± 11.7	1.93 (1.26–2.96)	0.003	1.65 (1.07–2.55)	0.023	1.58 (1.02–2.44)	0.039
Prevalent	22,241	67	123,722	54	60.6 ± 12.3	2.13 (1.59–2.86)	<0.001	2.08 (1.55–2.8)	<0.001	2.00 (1.49–2.70)	<0.001
**Total CD**							0.008		0.012		0.023
Control	37,893	19	185,637	10	64.9 ± 8.2	1 (Ref.)		1 (Ref.)		1 (Ref.)	
Case	12,631	15	61,416	24	53.7 ± 16.3	2.52 (1.28–4.95)		2.44 (1.22–4.86)		2.23 (1.12–4.45)	
**CD subgroup**											
Incident	5607	4	21,928	18	57.8 ± 16.2	1.71 (0.58–5.05)	0.332	1.52 (0.51–4.55)	0.457	1.36 (0.45–4.07)	0.587
Prevalent	7024	11	39,488	28	52.3 ± 16.9	3.05 (1.44–6.43)	0.004	3.12 (1.46–6.69)	0.004	2.91 (1.36–6.24)	0.006
**Total UC**							<0.001		<0.001		<0.001
Control	78,690	115	383,722	30	63.9 ± 10.5	1 (Ref.)		1 (Ref.)		1 (Ref.)	
Case	26,230	77	127,998	60	62.3 ± 10.6	2.00 (1.50–2.67)		1.92 (1.43–2.56)		1.85 (1.38–2.48)	
**UC subgroup**											
Incident	11,013	21	43,765	48	62.6 ± 11.0	1.97 (1.23–3.15)	0.005	1.75 (1.09–2.8)	0.020	1.68 (1.05–2.69)	0.031
Prevalent	15,217	56	84,234	66	62.2 ± 10.6	2.01 (1.46–2.77)	<0.001	1.99 (1.44–2.75)	<0.001	1.93 (1.40–2.67)	<0.001

CD, Crohn’s disease; CI, confidence interval; HR, hazard ratio; IBD, inflammatory bowel disease; No, number; PD, Parkinson’s disease; Ref., reference; UC, ulcerative colitis. * Mean ± SD. **^†^** Model 1: adjusted for age, sex. ^‡^ Model 2: adjusted for model 1 + place of residence, income level, diabetes mellitus, hypertension, dyslipidemia, depression, ischemic heart disease, history of myocardial infarction, and stroke. **^§^** Model 3: adjusted for model 2 + health care visits.

**Table 3:** Incidence and risk of Parkinson’s disease in patients with inflammatory bowel disease and non-inflammatory bowel disease controls according to medication.

	Total No.	PD Cases	Person-Years (y)	PD Incidence (/100,000 Person-Years)	Model 1 * HR (95% CI)	*P* Value	Model 2 ^†^ HR (95% CI)	*P* Value
**CD**								
Corticosteroids						0.006		<0.001
No	5214	11	26,582	41	1 (Ref.)		1 (Ref.)	
Yes	7417	4	34,834	11	0.19 (0.06–0.63)		0.08 (0.02–0.33)	
Immunomodulators						0.193		0.227
No	5623	13	27,465	47	1 (Ref.)		1 (Ref.)	
Yes	7008	2	33,951	6	0.35 (0.07–1.67)		0.38 (0.08–1.83)	
Anti-TNF						-		-
No	10,966	15	53,347	28	1 (Ref.)		1 (Ref.)	
Yes	1665	0	8069	0	-		-	
**UC**								
Corticosteroids						0.386		0.213
No	11,326	35	56,378	62	1 (Ref.)		1 (Ref.)	
Yes	14,904	42	71,621	59	0.82 (0.52–1.29)		0.75 (0.47–1.18)	
Immunomodulators						0.541		0.521
No	22,833	71	111,139	64	1 (Ref.)		1 (Ref.)	
Yes	3397	6	16,860	36	0.77 (0.33–1.78)		0.76 (0.33–1.76)	
Anti-TNF						-		-
No	25,785	77	126,117	61	1 (Ref.)		1 (Ref.)	
Yes	445	0	1881	0	-		-	
**Non-IBD controls**								
Corticosteroids						0.353		0.585
No	79,133	73	388,691	19	1 (Ref.)		1 (Ref.)	
Yes	37,450	61	180,669	34	1.18 (0.83–1.66)		1.10 (0.78–1.57)	
Immunomodulators						0.108		0.116
No	116,210	132	567,574	23	1 (Ref.)		1 (Ref.)	
Yes	373	2	1786	112	3.16 (0.78–12.84)		3.08 (0.76–12.52)	
Anti-TNF								-
No	116,555	134	569,235	24	1 (Ref.)		1 (Ref.)	
Yes	28	0	125	0	-		-	

CI, confidence interval; CD, Crohn’s disease; HR, hazard ratio; IBD, inflammatory bowel disease; No, number; PD, Parkinson’s disease; Ref., reference; TNF, tumor necrosis factor; UC, ulcerative colitis. * Model 1: adjusted for age, sex, place of residence, income level, diabetes mellitus, hypertension, dyslipidemia, depression, myocardial infarction, stroke, and ischemic heart disease. ^†^ Model 2: adjusted for model 1 + health care visits.

## Supplementary Materials

The following are available online at https://www.mdpi.com/2077-0383/8/8/1191/s1, Table S1: Risk of Parkinson’s disease among patients with inflammatory bowel diseases in a literature review, Table S2: Incidence and risk of Parkinson’s disease in patients with inflammatory bowel disease aged less than 60 years, Table S3: Incidence and risk of Parkinson’s disease in patients with inflammatory bowel disease aged 60 years and more.

## Abbreviations

CD	Crohn’s disease;
CI	confidence interval;
DM	diabetes mellitus;
HR	hazard ratio;
IBD	inflammatory bowel disease;
IL	interleukin;
ICD-10	International Classification of Diseases, tenth revision;
NHIS	National Health Insurance Service;
PD	Parkinson’s disease;
RID	rare intractable disease;
TNF	tumor necrosis factor;
UC	ulcerative colitis;
UK	United Kingdom.
