# Supplementary material for: Patients with Inflammatory Bowel Disease Are at an Increased Risk of Parkinson’s Disease: A South Korean Nationwide Population-Based Study
Source: J Clin Med. 2019 Aug 8;8(8):1191. doi: 10.3390/jcm8081191 (PMC6723604; doi:10.3390/jcm8081191)
Supplement: Supplementary file 1 [file jcm-08-01191-s001.zip › jcm_Supplementary table 1.docx]

**Table S1.** Risk of Parkinson's disease among patients with inflammatory bowel disease based on literature review

| **Author** | **Year of Publication** | **Country** | **Study design** | **Mean Age**  **(y)** | **No. of participants** | | **PD incidence**  **(/100,000 person-years)** | **HR/OR (95% CI)** | | | | **Adjusted by healthcare visits** |
| --- | --- | --- | --- | --- | --- | --- | --- | --- | --- | --- | --- | --- |
|  |  |  |  |  | IBD | Control |  | IBD | CD | UC | |  |
| Lin JC et al.[32] | 2016 | Taiwan | Cohort | 47 | 8373 | 33,492 | 174 | HR, 1.35  (1.08-1.68) | HR, 1.40  (1.11-1.77) | HR, 0.94  (0.49-1.84) | | No |
| Camacho-Soto A et al.[29] | 2018 | US | Case-control | NA | 4980 | 202,905 | NA | OR, 0.85  (0.80-0.91) | OR, 0.83  (0.74-0.93) | OR, 0.88  (0.82-0.96) | | Yes |
| Peter I et al.[33] | 2018 | US | Cohort | 51 | 144,018 | 720,090 | 73 | HR, 1.28  (1.14-1.44) | HR, 1.26  (1.03-1.53) | HR, 1.31  (1.14-1.51) | | No |
| Weimers P et al.[30] | 2018 | Sweden | Cohort | 45 | 39,652 | 396,520 | 40 | HR, 1.3  (1.0-1.6) | HR, 1.1  (0.7-1.7) | HR, 1.3  (1.0-1.7) | | No |
|  |  |  |  |  |  |  |  | HR, 0.9  (0.7-1.1) | HR, 0.9  (0.6-1.4) | | HR, 0.8  (0.6-1.1) | Yes |
| Villumsen M et al.[28,46] | 2018 | Denmark | Cohort | NA | 76,477 | 7,548,259 | 37 | HR, 1.22  (1.09-1.35) | HR, 1.12  (0.89-1.40) | HR, 1.35  (1.20-1.52) | | No |
|  |  |  |  |  |  |  |  | HR, 1.49  (1.34-1.66); | NA | NA | | Yes |
| Park S et al. | - | Korea | Cohort | 40 | 38,861 | 116,583 | 49 | HR, 1.95  (1.49-2.54) | HR, 2.44  (1.22-4.86) | HR, 1.92  (1.43-2.56) | | No |
|  |  |  |  |  |  |  |  | HR, 1.87  (1.43-2.44) | HR, 2.23  (1.12-4.45) | HR, 1.85  (1.38-2.48) | | Yes |

CI, confidence interval; CD, Crohn’s disease; HR, hazard ratio; IBD, inflammatory bowel disease; NA, not available; No, number; OR, odds ratio; PD, Parkinson’s disease; UC, ulcerative colitis.
