# Supplementary material for: Patients with Inflammatory Bowel Disease Are at an Increased Risk of Parkinson’s Disease: A South Korean Nationwide Population-Based Study
Source: J Clin Med. 2019 Aug 8;8(8):1191. doi: 10.3390/jcm8081191 (PMC6723604; doi:10.3390/jcm8081191)
Supplement: Supplementary file 1 [file jcm-08-01191-s001.zip › jcm_Supplementary table 2.docx]

**Table S2.** Incidence and risk of Parkinson’s disease in patients with inflammatory bowel disease aged less than 60 years

|  | **Total No.** | **PD cases** | **Person-years (y)** | **PD incidence (/100,000 person-years)** | **Model 1* HR**  **(95% CI)** | ***P* value** | **Model 2^†^ HR**  **(95% CI)** | ***P* value** | **Model 3**^‡^ **HR**  **(95% CI)** | ***P* value** |
| --- | --- | --- | --- | --- | --- | --- | --- | --- | --- | --- |
| **Total IBD** |  |  |  |  |  | < 0.001 |  | < 0.001 |  | < 0.001 |
| Control | 100,248 | 30 | 491,425 | 6 | 1 (Ref.) |  | 1 (Ref.) |  | 1 (Ref.) |  |
| Case | 33,416 | 30 | 163,584 | 18 | 3.00 (1.81-4.98) |  | 2.85 (1.71-4.75) |  | 2.63 (1.57-4.41) |  |
| **IBD subgroup** |  |  |  |  |  |  |  |  |  |  |
| Incident | 14,565 | 8 | 58,096 | 14 | 2.70 (1.23-5.91) | 0.013 | 2.47 (1.12-5.43) | 0.025 | 2.29 (1.04-5.06) | 0.039 |
| Prevalent | 18,851 | 22 | 150,497 | 15 | 3.13 (1.8-5.44) | < 0.001 | 3.02 (1.73-5.26) | < 0.001 | 2.79 (1.59-4.89) | < 0.001 |
| **Total CD** |  |  |  |  |  | < 0.001 |  | < 0.001 |  | 0.002 |
| Control | 35,763 | 4 | 175,809 | 2 | 1 (Ref.) |  | 1 (Ref.) |  | 1 (Ref.) |  |
| Case | 11,921 | 10 | 58,300 | 17 | 7.68 (2.41-24.47) |  | 7.70 (2.35-25.21) |  | 6.72 (2.02-22.39) |  |
| **CD subgroup** |  |  |  |  |  |  |  |  |  |  |
| Incident | 5,254 | 2 | 20,697 | 10 | 4.84 (0.88-26.64) | 0.070 | 4.75 (0.84-26.69) | 0.077 | 4.14 (0.73-23.62) | 0.110 |
| Prevalent | 6,667 | 8 | 37,603 | 21 | 8.99 (2.7-29.9) | < 0.001 | 9.09 (2.66-31.06) | < 0.001 | 7.95 (2.28-27.67) | 0.001 |
| **Total UC** |  |  |  |  |  | 0.005 |  | 0.010 |  | 0.020 |
| Control | 64,485 | 26 | 315,616 | 8 | 1 (Ref.) |  | 1 (Ref.) |  | 1 (Ref.) |  |
| Case | 21,495 | 20 | 105,294 | 19 | 2.30 (1.29-4.12) |  | 2.16 (1.2-3.88) |  | 2.02 (1.12-3.66) |  |
| **UC subgroup** |  |  |  |  |  |  |  |  |  |  |
| Incident | 9,311 | 6 | 37,399 | 16 | 2.36 (0.97-5.76) | 0.060 | 2.14 (0.87-5.25) | 0.097 | 2.02 (0.82-4.96) | 0.126 |
| Prevalent | 12,184 | 14 | 67,894 | 21 | 2.28 (1.19-4.37) | 0.013 | 2.16 (1.12-4.17) | 0.021 | 2.02 (1.04-3.92) | 0.037 |

CD, Crohn’s disease; CI, confidence interval; HR, hazard ratio; IBD, inflammatory bowel disease; No, number; PD, Parkinson’s disease; Ref., reference; UC, ulcerative colitis. *Model 1: adjusted for age, sex. ^†^Model 2: adjusted for model 1 + place of residence, income level, diabetes mellitus, hypertension, dyslipidemia, depression, ischemic heart disease, history of myocardial infarction, and stroke. ^‡^Model 3: adjusted for model 2 + healthcare visits.
