# Supplementary material for: Patients with Inflammatory Bowel Disease Are at an Increased Risk of Parkinson’s Disease: A South Korean Nationwide Population-Based Study
Source: J Clin Med. 2019 Aug 8;8(8):1191. doi: 10.3390/jcm8081191 (PMC6723604; doi:10.3390/jcm8081191)
Supplement: Supplementary file 1 [file jcm-08-01191-s001.zip › jcm_Supplementary table 3.docx]

**Table S3.** Incidence and risk of Parkinson’s disease in patients with inflammatory bowel disease aged 60 years and more

|  | **Total No.** | **PD cases** | **Person-years (y)** | **PD incidence (/100,000 person-years)** | **Model 1* HR**  **(95% CI)** | ***P* value** | **Model 2^†^ HR**  **(95% CI)** | ***P* value** | **Model 3‡ HR**  **(95% CI)** | ***P* value** |
| --- | --- | --- | --- | --- | --- | --- | --- | --- | --- | --- |
| **Total IBD** |  |  |  |  |  | < 0.001 |  | < 0.001 |  | 0.003 |
| Control | 16,335 | 104 | 77,935 | 133 | 1 (Ref.) |  | 1 (Ref.) |  | 1 (Ref.) |  |
| Case | 5,445 | 52 | 25,821 | 201 | 1.80 (1.31-2.47) |  | 1.68 (1.22-2.31) |  | 1.63 (1.19-2.24) |  |
| **IBD subgroup** |  |  |  |  |  |  |  |  |  |  |
| Incident | 2,055 | 17 | 7,596 | 224 | 1.74 (1.04-2.91) | 0.036 | 1.48 (0.88-2.49) | 0.138 | 1.42 (0.84-2.39) | 0.186 |
| Prevalent | 3,990 | 45 | 18,225 | 247 | 1.83 (1.29-2.59) | < 0.001 | 1.77 (1.24-2.52) | 0.002 | 1.72 (1.21-2.45) | 0.003 |
| **Total CD** |  |  |  |  |  | 0.885 |  | 0.975 |  | 0.898 |
| Control | 2,130 | 15 | 9,828 | 153 | 1 (Ref.) |  | 1 (Ref.) |  | 1 (Ref.) |  |
| Case | 710 | 5 | 3,116 | 160 | 1.02 (0.36-2.86) |  | 1.02 (0.36-2.86) |  | 0.93 (0.33-2.63) |  |
| **CD subgroup** |  |  |  |  |  |  |  |  |  |  |
| Incident | 353 | 2 | 1,231 | 163 | 0.88 (0.20-3.98) | 0.990 | 0.88 (0.20-3.98) | 0.872 | 0.80 (0.18-3.61) | 0.772 |
| Prevalent | 357 | 3 | 1,886 | 159 | 1.13 (0.32-3.99) | 0.849 | 1.13 (0.32-3.99) | 0.851 | 1.05 (0.30-3.75) | 0.938 |
| **Total UC** |  |  |  |  |  | < 0.001 |  | < 0.001 |  | < 0.001 |
| Control | 14,205 | 89 | 68,107 | 131 | 1 (Ref.) |  | 1 (Ref.) |  | 1 (Ref.) |  |
| Case | 4,735 | 57 | 22,705 | 251 | 1.92 (1.37-2.67) |  | 1.83 (1.31-2.56) |  | 1.79 (1.28-2.50) |  |
| **UC subgroup** |  |  |  |  |  |  |  |  |  |  |
| Incident | 1,702 | 15 | 6,365 | 236 | 1.89 (1.09-3.28) | 0.024 | 1.68 (0.97-2.93) | 0.066 | 1.62 (0.93-2.83) | 0.087 |
| Prevalent | 3,033 | 42 | 16,339 | 257 | 1.93 (1.33-2.78) | < 0.001 | 1.89 (1.31-2.75) | < 0.001 | 1.87 (1.28-2.69) | 0.001 |

CD, Crohn’s disease; CI, confidence interval; HR, hazard ratio; IBD, inflammatory bowel disease; No, number; PD, Parkinson’s disease; Ref., reference; UC, ulcerative colitis. *Model 1: adjusted for age, sex. ^†^Model 2: adjusted for model 1 + place of residence, income level, diabetes mellitus, hypertension, dyslipidemia, depression, ischemic heart disease, history of myocardial infarction, and stroke. ^‡^Model 3: adjusted for model 2 + healthcare visits.
